# Access to Neighborhood Amenities and Services and the Risk of 2-Year Nursing Home Placement Among Persons Living With Dementia

**DOI:** 10.1093/geroni/igaf011

**Published:** 2025-02-13

**Authors:** Yeon Jin Choi, Gillian Fennell, Jennifer A Ailshire

**Affiliations:** College of Social Work, University of Kentucky, Lexington, Kentucky, USA; Leonard Davis School of Gerontology, University of Southern California, Los Angeles, California, USA; Leonard Davis School of Gerontology, University of Southern California, Los Angeles, California, USA

**Keywords:** Aging in place, Caregiver support, Dementia-friendly community

## Abstract

**Background and Objectives:**

Older adults prefer to age in their homes and communities, but due to increased care needs associated with dementia, persons living with dementia are much more likely to move into nursing homes. Living in communities with greater access to neighborhood amenities and supports may reduce the risk of nursing home placement by helping persons living with dementia maintain their health and independence and lowering caregiving burden and stress. This study aims to identify neighborhood amenities and services that are associated with nursing home transitions among persons living with dementia.

**Research Design and Methods:**

We used data from the 2004–2016 waves of the Health and Retirement Study (HRS), which includes 3 507 older adults with dementia, linked with the HRS Contextual Data Resource and the National Neighborhood Data Archive. Logistic regression models were estimated adjusting for sociodemographic and neighborhood characteristics.

**Results:**

Findings suggest that access to more park areas, healthy food outlets, and home health services was associated with a lower risk of 2-year nursing home placement.

**Discussion and Implications:**

These findings emphasize the importance of neighborhood amenities and services as essential components of supportive communities, enabling persons living with dementia to age in place.

**Translational significance:** In light of rising dementia prevalence and the strong preference for aging in place, our study identified neighborhood amenities and services that can support persons living with dementia to avoid nursing home placement. Our findings indicate that greater availability of park areas, healthy food outlets, and home health services was associated with a lower risk of 2-year nursing home placement. This work emphasizes the importance of developing supportive communities for persons living with dementia, particularly in the built and service environment domains.

The majority of older Americans prefer to remain in their homes, or “age in place,” rather than move into institutionalized care (1) in order to maintain autonomy and stay socially active (2). However, aging in place may not be feasible for some older adults—especially those with physical and cognitive impairments. In fact, among the strongest predictors of institutionalization are individuals’ physical and cognitive function as well as the quality of life of their family members who serve as caregivers (3,4).

Dementia limits individuals’ capacity to perform activities of daily living (ADLs; eating, toileting, etc.) and instrumental activities of daily living (IADLs; managing money, preparing meals, etc.), significantly reducing their abilities to remain independent (5,6). Impairment of daily tasks at this level requires a higher intensity of care translating to a greater caregiver burden (7). Relative to non-dementia caregivers, dementia caregivers exert more physical effort while providing care (7). Behavioral and psychological symptoms of dementia, such as agitation, aggression, delusions, and wandering, are another primary contributors to caregiver burden (8). Beyond burden and stress, caregiver burnout—or a state of whole exhaustion stemming from providing care to another individual—is far more common among dementia caregivers (~25%–40%), leading to a greater need to move care recipients into institutionalized care settings (9–11). Because of this, institutionalization rates are disproportionately high among persons living with dementia; compared to only 2% for the total population over age 65, 17% of individuals with dementia transition to nursing homes (12,13).

Due to the increasing prevalence of dementia (14) and the strong preference for aging in place (1), there has been a growing effort to define “dementia-friendly communities.” Dementia-friendly communities have been described as places in which persons living with dementia can feel safe finding their way around, have access to necessary and familiar services, and maintain social connections with compassionate community members (15,16). The concept of dementia-friendly communities emulates the core insight from the person–environment fit theory (17,18), which posits that older adults’ well-being is dependent on the fit or interaction between their needs and environment. Based on this theory, individuals living with dementia may still be able to maintain their functioning and successfully age in place if they are accommodated in an environment that adequately addresses their needs. Various domains, including the physical, social, and service environment, can be leveraged to create supportive environments for persons living with dementia. However, there is a paucity of research identifying neighborhood amenities within the built and service environment that can enable persons living with dementia to remain in their homes and communities.

## Neighborhood Resources and Health

Ample evidence underscores the importance of neighborhood resources in supporting community-dwelling older adults maintain independence and enable aging in place. Neighborhood resources include amenities—features, facilities, or services within a neighborhood that enhance residents’ quality of life. Previous studies have reported that living in resource-rich neighborhoods, or neighborhoods with greater access to green spaces, healthy foods, and recreation centers, was associated with a lower risk of obesity, heart disease, and functional limitations (19,20). Other studies have also found that the presence of local community centers, retail stores, and restaurants was associated with better cognitive function and slower cognitive decline (21,22). In fact, older residents and community service providers have cited the availability of these amenities and supportive services as factors enabling individuals to age in place (23,24).

Although there is limited research, previous studies have shown that neighborhood amenities (e.g., parks, libraries, museums) can provide meaningful benefits for persons living with dementia and their caregivers through accessible community programming. For example, walking groups in parks, and library/museum programs that focus on art, storytelling, or engaging with music have elicited noticeable improvements in socialization, self-esteem, and quality of life for persons living with dementia (25–27). These activities also contribute to short-term improvements in memory, new learning, verbal communication, mood, and sustained attention (25,27,28). These documented improvements show potential for neighborhood and community programming to slow the rate of cognitive decline in persons living with dementia and mitigate some of the disruptive behavioral symptoms of dementia that contribute to transitions into nursing homes.

Local stores provide places for persons living with dementia and their caregivers to recognize familiar faces and engage socially with their community, which may help reduce feelings of agitation and isolation for both parties. For caregivers, strong community ties and social resources for persons living with dementia offer much needed support and respite care (29,30). Home health services can also provide a similar benefit in reducing caregiver burden (31,32).

Based on previous findings, access to neighborhood amenities and services may help maintain the independence of persons living with dementia and mitigate caregiver burden, thereby reducing the risk of institutionalization. However, despite the potentially important role of neighborhood resources in supporting persons living with dementia to age in place, no study has investigated the relationship between the availability of neighborhood amenities and services and the risk of institutionalization. To address this gap, this study aims to identify supportive neighborhood amenities and services that can prevent or delay nursing home placement of persons living with dementia, utilizing a national sample of community-dwelling older Americans with dementia. We hypothesized that greater access to or availability of neighborhood amenities and services will be associated with the decreased risk of nursing home placement of older adults living with dementia. The amenities we expect to correlate with a decreased risk of nursing home placement in this population include park areas, healthy food outlets, social and cultural amenities, retail stores, social services for older adults and people with disabilities, and home health services. The findings of this study will deepen our understanding of key features of supportive built and service environments for older adults living with dementia.

## Method

### Data and Sample

We used data from the Health and Retirement Study (HRS; https://hrs.isr.umich.edu/), a nationally representative, longitudinal study of community-dwelling adults over age 50 in the United States. The HRS has conducted biennial surveys since 1992. For this study, we used data from the 2004–2016 waves of the HRS as neighborhood data were available from 2003 to 2017. The neighborhood data from the HRS Contextual Data Resource (HRS-CDR) and the National Neighborhood Data Archive (NaNDA; https://nanda.isr.umich.edu/) were linked to the HRS via respondents’ residential addresses geocoded to U.S. census tracts. The HRS-CDR is a collection of datasets covering a variety of area measures, such as neighborhood demographic characteristics, food environments, and healthcare access (33). Among the available datasets, we used census tract-level food access data and neighborhood demographic data created using the United States Department of Agriculture (USDA) Food Access Research Atlas and the Decennial Census and American Community Survey. We also obtained data on other neighborhood amenity measures and neighborhood socioeconomic status from the NaNDA, which is a publicly available data archive containing nationwide measures of the physical and social environment at various levels of geography, including at the county and census tract levels (34). Additionally, we linked HRS respondents with state-level Medicaid home and community-based services (HCBS) expenditure from the Centers for Medicare & Medicaid Services.

We limited our analytic sample to cohort-eligible respondents with dementia who lived in the community. The HRS assesses respondents’ cognitive function with a range of tests adapted from the Telephone Interview for Cognitive Status. Following previous work (35,36), we used a 27-point cognitive scale that includes total word recall (0–20 points), serial 7’s test (0–2 points), and backwards counting (0–5 points) to determine dementia status. Individuals were classified as having cognitive impairment consistent with dementia if they had a score of 0–6 (36). For respondents who did not take the cognitive assessment, we used a score composed of proxy assessments of respondents’ memory (range: 0 excellent–4 poor) and IADL difficulties, as well as the interviewer’s assessment of the respondents’ difficulty completing the interview (range: 0 none–2 severe). Scores on the proxy and interviewer assessment ranged from 0 to 11, and scores of 6–11 indicated impairment in cognitive functioning consistent with dementia (35). To reduce measurement error in dementia status, individuals were determined to have confirmed dementia if they reported dementia for 2 consecutive waves (37). Most respondents with confirmed dementia consistently reported low cognitive functioning. Approximately 1.5% of respondents exhibited temporary improvements, though these improvements were not sustained.

**Figure 1.**
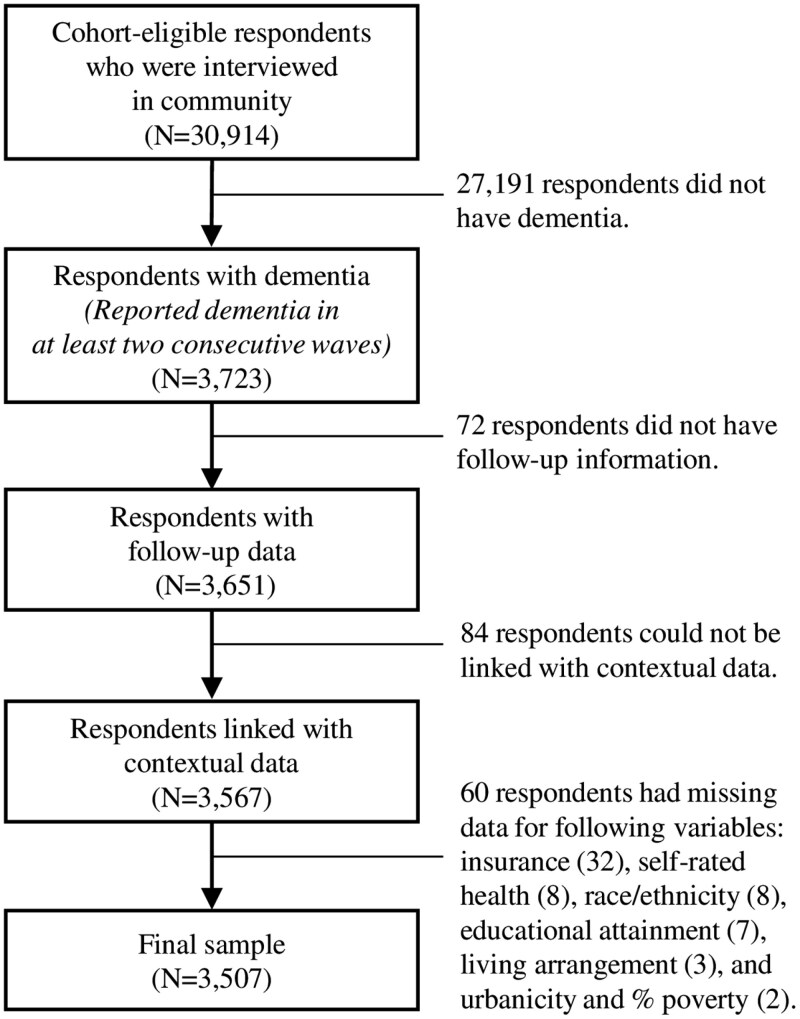
Sample inclusion criteria.

The 2004–2016 HRS includes 3 723 community-dwelling, cohort-eligible respondents classified as having dementia. We omitted 72 respondents who did not have follow-up data, 84 respondents who could not be linked with contextual data, and 60 respondents with missing data on key variables and covariates. The final analytic sample includes 3 507 respondents (Figure 1).

### Measures

#### Nursing home transition.

We examined whether respondents (1) remained in the community, (2) transitioned into a nursing home, or (3) died in or before the following wave. While the transition into a nursing home is the primary interest of this study, we included death as a competing risk to account for respondents who were not at risk of nursing home placement.

#### Neighborhood amenities and services.

We included 6 neighborhood measures: park area, food access, social and cultural amenities, retail stores, social services for older adults and people with disabilities, and home health services. *Park area* is a tract-level variable with 3 categories: (1) no park, (2) park area covering less than 12.5% of the tract area, and (3) park area covering 12.5% or more of the tract area (38). *Food access*—access to healthy food—is a tract-level variable defined based on the USDA definition of food desert (ie, low-income, low-access tract) (39). All other domains are continuous variables created by summing the number of relevant organizations and service providers per square mile. *Social and cultural amenities* (county-level) include art performance organizations, museums, historical sites, and libraries. *Retail stores* (tract-level) include department stores and other retail stores. *Social services for older adults and people with disabilities* (county-level) include senior centers and adult day care centers, and *home health services* (county-level) include agencies providing in-home personal care and visiting nurses. We summed the number of social and cultural amenities, social services, and home health services at the county-level, given their limited numbers. We then divided the total by the number of adults aged 65 and older in the geographic area, and top-coded at the 99th percentile to correct for skewness in the data.

#### Covariates.

Individual-level covariates included age (in years; range: 50–109), sex (male [reference]; female), race/ethnicity (non-Hispanic White [reference]; non-Hispanic Black; non-Hispanic other, Hispanic), education (less than high school [reference]; high school; more than high school), household assets (converted to decimal; range: 1–10), health insurance (no insurance [reference]; health insurance; long-term care insurance), living arrangements (living alone [reference]; living with a spouse; living with children; living with relatives; living with unrelated adults), marital status (married/partnered [reference]; separated/divorced; widowed; never married), self-rated health (range: 1–5 excellent), number of caregivers (top-coded at 99th percentile; range: 0–5), and hours of care received (less than 14 hours [reference]; 14 hours or more). Neighborhood-level covariates included the number of nursing homes in a county (divided by the number of older population in the county, then top-coded at 99th percentile; range: 0–15.26), urbanicity (urban [reference]; rural), the percentage of population with income below poverty level in a tract (range: 0–1), and the state-level Medicaid HCBS spending per person (in dollars; range: 21.39–5477.74).

### Analysis Plan

We first estimated sample characteristics. We then tested whether greater access to neighborhood resources was negatively associated with nursing home placement using multinomial logistic regression. The reference outcome in our models was “staying in the community.” Alternatively, persons living with dementia could either be observed at follow-up after 2 years as deceased or as having transitioned into a nursing home. On average, respondents were observed for 2 waves after the confirmation of dementia status, but some respondents were observed for as many as 7 waves. The percentage of respondents observed for one wave was 41.9%, for 2 waves was 23.5%, for 3 waves was 14.3%, for 4 waves was 8.0%, for 5 waves was 5.1%, for 6 waves was 3.8%, and for 7 waves was 3.5%. Neighborhood amenities and services were included separately in Model 1 through Model 6, controlling for all individual- and neighborhood-level covariates. We reported cluster-adjusted standard errors to account for repeated within-person observations. To ensure population-level representation of community-dwelling adults aged 50 and over in the United States, the HRS provides sample weights for each wave. These weights account for the differential probabilities of selection, non-response, and post-stratification adjustments to align with the age, gender, racial/ethnic, and socioeconomic distribution of the U.S. population aged 50 and older (40,41). We used wave-specific sample weights for each respondent. Analyses were conducted using Stata 17.

## Results

### Sample Characteristics

Table 1 presents sample characteristics. The majority of the sample remained in the community (62.4%), while 6.2% transitioned into a nursing home, and the remaining 31.5% died before or in the following wave. In general, respondents who remained in the community in the following wave had the highest access to neighborhood amenities and services: 6.2% lived in a tract with more park area (≥12.5% of tract) and 32.3% had high access to healthy food. By comparison, those who transitioned into a nursing home had the lowest access: 4.6% of them lived in a tract with more park area, and 27.4% had high access to healthy food. Those who remained in the community also lived in communities with more social and cultural amenities, social services, and home health services than the other groups.

**Table 1.** Sample Characteristics (*N* = 3 507)

Characteristics	Whole Sample	Stayed in Community	Institutionalized	Deceased
	*M* (*SD*)/%	*M* (*SD*)/%	*M* (*SD*)/%	*M* (*SD*)/%
Transition				
Stayed in community	62.4%	100%		
Institutionalized	6.2%		100%	
Deceased	31.5%			100%
Tract-level park area				
No park	29.1%	28.7%	26.9%	30.2%
Less than 12.5%	64.9%	65.1%	68.5%	63.9%
12.5% or more	6.0%	6.2%	4.6%	5.9%
Tract-level food access				
Low	68.0%	67.7%	72.6%	67.6%
High	32.0%	32.3%	27.4%	32.4%
Social/cultural amenities in county^a^^,^^b^	4.38 (8.14)	4.55 (8.54)	3.5 (6.17)	4.22 (7.69)
Retail stores in tract^a^^,^^b^	5.09 (14.75)	5.27 (15.13)	3.93 (11.33)	4.97 (14.62)
Social services in county^a^^,^^b^	0.89 (1.61)	0.93 (1.70)	0.78 (1.19)	0.84 (1.52)
Home health services in county^a^^,^^b^	1.07 (1.65)	1.13 (1.73)	0.85 (1.21)	1.01 (1.57)
Age (range: 50–109)	76.7 (11.23)	74.0 (11.1)	82.2 (8.4)	80.8 (10.3)
Sex				
Male	42.1%	41.7%	26.5%	46.0%
Female	57.9%	58.4%	73.5%	54.0%
Race/ethnicity				
Non-Hispanic White	54.4%	45.9%	80.9%	66.2%
Non-Hispanic Black	24.4%	28.7%	11.3%	18.7%
Non-Hispanic other	3.9%	4.5%	2.6%	2.9%
Hispanic	17.2%	20.9%	5.3%	12.2%
Education				
Less than high school	59.2%	63.3%	44.3%	54.1%
High school	24.3%	22.5%	33.7%	25.9%
More than high school	16.6%	14.3%	22.0%	20.0%
Household asset (range: 1–10)	3.8 (2.7)	3.5 (2.6)	4.6 (2.7)	4.2 (0.1)
Insurance				
No insurance	4.0%	5.5%	0.6%	1.6%
Health insurance	88.9%	88.4%	87.3%	90.1%
Long-term care insurance	7.2%	6.1%	12.1%	8.4%
Living arrangement				
Living alone	28.4%	26.6%	48.4%	28.1%
Living with a spouse	46.6%	49.2%	32.5%	44.2%
Living with children	16.0%	15.3%	12.2%	18.3%
Living with relatives	6.8%	7.0%	5.9%	6.6%
Living with unrelated adults	2.1%	1.9%	0.9%	2.8%
Marital status				
Married/partnered	40.5%	41.5%	28.1%	40.8%
Separated/divorced	14.6%	16.8%	11.2%	10.9%
Widowed	39.5%	35.4%	56.0%	44.4%
Never married	5.5%	6.4%	4.7%	3.9%
Self-rated health (range: 1–5 excellent)	2.4 (1.1)	2.5 (1.1)	2.5 (1.1)	2.2 (1.1)
Number of caregivers (range: 0–5)	0.9 (1.2)	0.7 (1.1)	1.1 (1.1)	1.1 (1.3)
Hours of caregiving per week				
Less than 14 h	81.0%	86.2%	77.8%	71.3%
14 h or more	19.0%	13.8%	22.2%	28.7%
Number of nursing home in county^a^^,^^b^	2.1 (2.9)	2.1 (3.0)	1.9 (2.2)	2.0 (2.8)
Urbanicity				
Urban	74.3%	73.3%	72.9%	76.6%
Rural	25.7%	26.7%	27.2%	23.4%
% population in poverty in tract (range: 0–1)	0.19 (0.1)	0.21 (0.1)	0.14 (0.1)	0.17 (0.1)
State-level Medicaid HCBS spending^b^	1156.3 (914.0)	1152.5 (907.3)	1193.1 (947.4)	1156.6 (917.8)

*Notes*: HCBS = home and community-based services; *M* = mean; *SD* = standard deviation.

^a^Number per square mile. Top-coded at 99th percentile.

^b^Adjusted for the older population (age 65 and over) in the geographic area.

### Neighborhood Amenities and Services and the Risk of Institutionalization

We present the models predicting the relative risk of a 2-year transition to a nursing home in Table 2. Models 1 through 6 assessed the association of a single neighborhood resource with the relative risk of 2-year nursing home placement while additionally controlling for individual, household, and community characteristics. Tract-level park area was significantly associated with a lower risk of nursing home placement (12.5% or more: relative risk ratio [RRR] = 0.56, *p* < .05). Living in a tract with high food access was significantly associated with a 25% lower likelihood of moving to a nursing home (RRR = 0.75, *p* < .05). The number of social and cultural amenities was not statistically significantly associated with the risk of nursing home placement (RRR = 0.96; *p* = .80). In the remaining 3 models, presence of retail stores and social services was not significantly associated with nursing home placement risk (retail store: RRR = 1.00, *p* = .71; social services: RRR = 0.88, *p* = .26), while one additional home health services per square mile within a county corresponded to a 24% reduced risk of institutionalization (RRR = 0.76, *p* < .01).

**Table 2.** Multinomial Logistic Regressions Estimating the Risk of Transitioning to a Nursing Home Relative to Aging in Place (*N* = 3 507)

Variable	Model 1: Park Area		Model 2: Food Access			Model 3: Social/Cultural Amenities			Model 4: Retail Stores			Model 5: Social Services			Model 6: Home Health		
	RRR	*SE*	RRR		*SE*	RRR		*SE*	RRR		*SE*	RRR		*SE*	RRR		*SE*
Tract-level park area (ref: no park)																	
Less than 12.5%	1.04	0.14															
12.5% or more	0.56*	0.17															
Tract-level food access (ref: low)																	
High			0.75*		0.11												
Social/cultural amenities in county^a,b^						0.96		0.02									
Retail stores in tract^a,b^									1.00		0.01						
Social services in county^a,b^												0.88		0.10			
Home health services in county^a,b^															0.76**		0.08
Age	1.06***	0.01	1.06***		0.01	1.06***		0.01	1.06***		0.01	1.06***		0.01	1.06***		0.01
Sex (ref: male)																	
Female	1.45***	0.21	1.44*		0.21	1.45*		0.21	1.46*		0.21	1.46*		0.21	1.45*		0.21
Race/ethnicity (ref: non-Hispanic White)																	
Non-Hispanic Black	0.35*	0.07	0.35***		0.07	0.37***		0.07	0.36***		0.07	0.36***		0.07	0.37***		0.07
Non-Hispanic other	0.68	0.26	0.69		0.26	0.69		0.27	0.68		0.26	0.67		0.26	0.69		0.27
Hispanic	0.22	0.06	0.22***		0.06	0.24***		0.06	0.23***		0.06	0.23***		0.06	0.25***		0.06
Education (ref: less than high school)																	
High school	1.42	0.21	1.39*		0.20	1.42*		0.21	1.40*		0.20	1.40*		0.20	1.42*		0.21
More than high school	1.36	0.24	1.32		0.23	1.38		0.24	1.36		0.24	1.35		0.23	1.39		0.24
Household asset (decimal; range: 1–10)	0.99	0.02	0.99		0.02	0.99		0.02	0.99		0.24	0.99		0.02	0.99		0.02
Insurance (ref: no insurance)																	
Health insurance	1.81	1.18	1.81		1.19	1.76		1.15	1.79		1.17	1.79		1.17	1.73		1.13
Long-term care insurance	2.80	1.92	2.85		1.96	2.77		1.90	2.82		1.94	2.83		1.94	2.74		1.88
Living arrangement (ref: living alone)																	
Living with a spouse	0.95	0.95	0.97		0.34	0.97		0.34	0.96		0.33	0.97		0.33	0.98		0.34
Living with children	0.38***	0.38	0.39***		0.07	0.39***		0.07	0.38***		0.07	0.38***		0.07	0.39***		0.07
Living with relatives	0.64	0.64	0.65		0.16	0.64		0.15	0.64		0.15	0.65		0.16	0.64		0.15
Living with unrelated adults	0.20**	0.20	0.21**		0.11	0.21**		0.11	0.20**		0.11	0.20**		0.11	0.21**		0.11
Marital status (ref: married/partnered)																	
Separated or divorced	1.95*	0.63	2.00*		0.65	1.99*		0.64	1.97*		0.64	1.98*		0.64	2.00*		0.65
Widowed	1.86	0.67	1.90		0.69	1.91		0.68	1.88		0.67	1.91		0.68	1.92		0.68
Never married	3.05*	1.46	3.16*		1.57	3.10*		1.49	3.07*		1.47	3.10*		1.49	3.17*		1.52
Number of caregivers (range: 0–5)	1.15**	0.06	1.16**		0.60	1.16**		0.06	1.16**		0.60	1.16**		0.06	1.16		0.06
Self-rated health (range: 1 poor–5 excellent)	0.96	0.05	0.97		0.50	0.97		0.05	0.97		0.05	0.97		0.05	0.96*		0.05
Hours of care received per week (ref: <14 h)																	
14 h or more	1.44*	0.26	1.43*		0.25	1.45*		0.26	1.43*		0.25	1.43*		0.25	1.44*		0.25
Urbanicity (ref: urban)																	
Rural	1.12	0.17	1.23		0.19	1.17		0.18	1.15		0.17	1.17		0.18	1.15		0.17
Number of nursing home in county^a,b^	1.03	0.03	1.04		0.03	1.14*		0.06	1.03		0.03	1.10		0.07	1.18**		0.06
% population in poverty in tract (range: 0–1)	0.12**	0.08	0.12**		0.08	0.12**		0.08	0.13**		0.09	0.12**		0.08	0.12**		0.08
State-level Medicaid HCBS spending^b^	1.00	0.00	1.00		0.00	1.00		0.00	1.00		0.00	1.00		0.00	1.00		0.00
Constant	0.00***	0.00	0.00***		0.00	0.00***		0.00	0.00***		0.00	0.00***		0.00	0.00***		0.00

*Notes*: HCBS = home and community-based services; ref = reference; RRR = relative risk ratio; *SE* = standard error.

^a^Number per square mile. Top-coded at 99th percentile. ^b^Adjusted for the older population (age 65 and over) in the geographic area. * *p* < .05; ** *p* < .01; *** *p* < .001.

The models predicting the relative risk of dying are presented in Supplementary Table 1.

### Sensitivity Analysis

There are few published guidelines for the appropriate amount of park area per capita or per unit of area (eg, square mile). Therefore, we adopted a conservative cut point of 12.5%, as suggested by Moeller (38). Notably, most tract areas in our analysis (90.8%) had less than 12.5% park area. To ensure the robustness of our findings for the association between park area and the risk of institutionalization, we conducted additional analyses using lower cut points and found that the association remained consistent to a cut point of 5.5% or more park area (see Supplementary Table 2).

## Discussion

As the number of persons living with dementia is projected to increase, there has been a growing interest in the potential for dementia-friendly communities to promote independence among persons living with dementia, reduce strain on their caregivers, and enable aging in place. While efforts have been made to understand the characteristics of dementia-friendly communities in some countries, neighborhood resources that affect persons living with dementia in the United States have received less attention. To bridge this gap, this study explored neighborhood amenities that can support persons living with dementia in a national sample, grounded in the person–environment fit theory (17,18). This study is among the first to identify specific neighborhood amenities and services that are associated with a reduced risk of nursing home placement among persons living with dementia in the United States. It highlights potential key features of supportive communities that enable persons living with dementia to engage socially, enjoy a high quality of life, and live with a fair amount of independence (15).

We found that more park area was associated with a reduced risk for transition into a nursing home among persons living with dementia. Neighborhood green spaces have been found to be associated with the health and well-being of older adults (20,22,42), likely because green spaces increase engagement in physical activity, provide places to socialize, and promote quality of life (43,44). Our finding suggests that green spaces may also benefit persons living with dementia and their caregivers. Recent studies suggest that physical activity—a common correlate of neighborhood green spaces (43,45)—can improve physical and cognitive function and reduce behavioral and psychological symptoms of dementia (46,47). In addition, by providing opportunities for recreation and social interaction (45), parks can help persons living with dementia retain cognitive function and promote caregivers’ physical and mental health (48,49). Access to more park areas also reduced the risk of dying, which is congruent with emerging research highlighting the negative association between green space and all-cause mortality (50). Together, our findings suggest that park space is an important facet of supportive communities for older adults living with dementia and their caregivers.

Consistent with our expectations, greater access to healthy food was also significantly associated with a lower risk of nursing home placement. Previous studies have reported that access to grocery stores offers opportunities to eat nutritiously (51), which has been found to promote health and improve cognitive function (52,53). The importance of healthy food access is also relevant to dementia caregivers. The convenience of local healthy food may reduce caregiver burden and risk of food insecurity, which can pose a threat to their own health and impair their abilities to continue providing in-home care to persons living with dementia (54). Our data did not allow us to describe the prevalence of food insecurity in caregivers explicitly, but two-thirds of our sample had low access to healthy food, which can be extrapolated to their caregivers. This is consistent with prior work showing a high prevalence of food insecurity and undernutrition among persons living with dementia and their caregivers (55,56), suggesting that food access may be an important area of intervention that can promote the health and well-being of this population.

The presence of local social and cultural hubs (ie, libraries, museums, arts facilities) was not statistically significantly associated with the risk of nursing home placement in our sample. Previous studies have reported that dementia-focused programs and events offered at social and cultural hubs contribute to promoting cognitive function, mood, and well-being of persons living with dementia by providing opportunities for cognitive stimulation and social interaction (25,27,28). Previous studies also noted reduced stress and feelings of social isolation among caregivers who participated in such programs and events (28,57). The lack of an association in our study may be due to limitations in the data we used, which provides the number of social and cultural hubs in a geographic area, but no information about the programs and events these facilities offer. Further research is needed to investigate the importance of local social and cultural hubs as a feature of supportive neighborhoods for older adults living with dementia.

Our results suggest that the number of tract-level retail stores was also not statistically significantly associated with 2-year nursing home placement in our sample. This may be due to the limited types of retail stores included in this study or the increasing dependency on online shopping. In a recent survey conducted by AARP (58), despite their preference for in-store shopping, caregivers turn to online shopping to avoid inconvenience and difficulties associated with shopping with their family members in need of care. While retail stores may not be a crucial feature of supportive communities for older adults living with dementia, in-store shopping has been found to provide cognitive stimulation, socialization, and physical activity for the population (58). Business owners and local governments should consider ways to accommodate needs and improve shopping experiences for persons living with dementia and their caregivers.

We found that the availability of social services was not statistically significantly associated with a reduced risk of nursing home placement. This conflicts with prior work suggesting that senior centers and adult day care centers promote social engagement and mental stimulation, which can delay cognitive decline in persons living with dementia (59) and reduce caregiving burden and stress (60,61). This inconsistency may be due to limitations in our measure, which aggregated the number of social service organizations offering services for older adults and people with disabilities. Thus, this variable may have included services that were not relevant to persons living with dementia and their caregivers, such as disability support groups. Additionally, the variable did not provide detailed information on the type of services and programs these organizations offer, making it difficult to assess its relevance to our sample. Further research using a more refined measure would enhance our understanding of how social services support older adults living with dementia to age in place.

As expected, the availability of home health services was associated with a significant reduction in the risk of transition into a nursing home. This finding is consistent with prior work on a sample of older adults discharged from skilled nursing facilities (SNFs) (62,63). More specifically, 90 days after SNF discharge, the receipt of home health services reduced patients’ odds of death by 37% (62) and doubled the amount of time persons living with dementia remained in the home instead of being re-admitted (63). This may be due to the comprehensive support provided by home health services, including skilled nursing, therapy, and personal care, which improve the well-being of persons living with dementia and their caregivers through medical and respite care. Despite these potential benefits, lack of knowledge of local services, limited availability, access issues (eg, lack of transportation or financial resources), and negative perceptions of services have been identified as barriers to service utilization among persons living with dementia and their caregivers (64,65). Therefore, while the provision of social and home health services is essential for older adults living with dementia, the availability of these services alone is insufficient to ensure their access and utilization. Without access to public transportation, financial support, and the elimination of stigma and mistrust related to home health services, their benefits will be less apparent.

The general notion that amenity *availability* does not guarantee *utilization* is an important one within this population. Persons with dementia often experience a “shrinking world,” characterized by less frequent and more selective out-of-home trips. Margot-Cattin et al. (66) found that individuals visit retail stores, places of social, cultural, and spiritual significance, and recreation centers less frequently following a dementia diagnosis. Instead, those with dementia are more likely to venture out for medical care, and to places where they can connect with nature (eg, parks). This prior work (66) corroborates and sheds additional light on our findings regarding the varying influences of neighborhood amenities and services on the risk of institutionalization among this vulnerable group.

### Limitations

This study has several limitations. First, the contextual data used in this study provide information on the availability of neighborhood amenities and services within a tract or county, rather than their proximity to the respondent’s home, which may not accurately capture the respondents’ accessibility. In addition, we do not have information about whether and how persons living with dementia and their caregivers use neighborhood amenities and services. Although there are a number of studies reporting a correlation between access to services and utilization (67), the availability of neighborhood resources may not reflect actual utilization due to mobility, affordability, or lack of knowledge.

## Conclusion

Our study highlights the importance of neighborhood resources, including park areas, food access, and home health services, in enabling persons living with dementia to age in place. These findings provide empirical evidence supporting the significance of person–environment fit in this population, specifically within built and service environment domains, and offer valuable implications for individuals living with dementia and their caregivers. Based on the findings, policymakers and practitioners aiming to support people living with dementia and their families should prioritize increasing access to and encouraging the utilization of these amenities and services. While this study focused on the availability of neighborhood amenities in the built and service environments, future research on more diverse aspects, such as social environments, would contribute to establishing a holistic community model for older adults living with dementia and their families.

## Supplementary Material

igaf011_suppl_Supplementary_Materials

## Data Availability

This study was not preregistered. This study used publicly available data from the Health and Retirement Study (HRS), HRS Contextual Data Resource, the National Neighborhood Data Archive, and the Centers for Medicare & Medicaid Services. The original data can be requested and downloaded at the following websites: https://hrs.isr.umich.edu/; https://nanda.isr.umich.edu/; and https://www.medicaid.gov/

## References

[1] Binette J. 2021 *Home and Community Preference Survey: A National Survey of Adults Age 18-Plus*. AARP Research; 2021. https://doi.org/10.26419/res.00479.001

[2] Wiles JL , Leibing A, Guberman N, Reeve J, Allen RES. The meaning of “aging in place” to older people. Gerontologist. 2012;52(3):357–366. https://doi.org/10.1093/geront/gnr098 21983126

[3] Argimon JM , Limon E, Vila J, Cabezas C. Health-related quality-of-life of care-givers as a predictor of nursing-home placement of patients with dementia. Alzheimer Dis Associated Disorders. 2005;19(1):41–44. https://doi.org/10.1097/01.wad.0000160343.96562.8e 15764871

[4] Vandepitte S , Putman K, Van Den Noortgate N, et al Factors associated with the caregivers’ desire to institutionalize persons with dementia: a cross-sectional study. Dement Geriatr Cogn Disord. 2018;46(5-6):298–309. https://doi.org/10.1159/000494023 30453298

[5] Cipriani G , Danti S, Picchi L, Nuti A, Fiorino MD. Daily functioning and dementia. Dement Neuropsychol. 2020;14:93–102. https://doi.org/10.1590/1980-57642020dn14-020001 32595877 PMC7304278

[6] Prizer LP , Zimmerman S. Progressive support for activities of daily living for persons living with dementia. Gerontologist. 2018;58(suppl_1):S74–S87. https://doi.org/10.1093/geront/gnx103 29361063 PMC5881654

[7] Parker LJ , Fabius C, Rivers E, Taylor JL. Is dementia-specific caregiving compared with non-dementia caregiving associated with physical difficulty among caregivers for community-dwelling adults? J Appl Gerontol. 2022;41(4):1074–1080. https://doi.org/10.1177/07334648211014352 34041929 PMC8664093

[8] Chiao CY , Wu HS, Hsiao CY. Caregiver burden for informal caregivers of patients with dementia: a systematic review: caregiver burden for informal caregivers. Int Nurs Rev. 2015;62(3):340–350. https://doi.org/10.1111/inr.12194 26058542

[9] Eska K , Graessel E, Donath C, Schwarzkopf L, Lauterberg J, Holle R. Predictors of institutionalization of dementia patients in mild and moderate stages: a 4-year prospective analysis. Dement Geriatr Cogn Dis Extra. 2013;3(1):426–445. https://doi.org/10.1159/000355079 24348504 PMC3843910

[10] Truzzi A , Valente L, Ulstein I, Engelhardt E, Laks J, Engedal K. Burnout in familial caregivers of patients with dementia. Braz J Psychiatry. 2012;34(4):405–412. https://doi.org/10.1016/j.rbp.2012.02.006 23429811

[11] Yıldızhan E , Ören N, Erdoğan A, Bal F. The burden of care and burnout in individuals caring for patients with Alzheimer’s disease. Community Ment Health J. 2019;55(2):304–310. https://doi.org/10.1007/s10597-018-0276-2 29680976

[12] Harrison KL , Ritchie CS, Patel K, et al Care settings and clinical characteristics of older adults with moderately severe dementia. J Am Geriatr Soc. 2019;67(9):1907–1912. https://doi.org/10.1111/jgs.16054 31389002 PMC6732035

[13] National Center for Health Statistics. Nursing Home Care. Centers for Disease Control and Prevention; 2022. Accessed October 3, 2023. https://www.cdc.gov/nchs/fastats/nursing-home-care.htm

[14] Nichols E , Steinmetz JD, Vollset SE, et al Estimation of the global prevalence of dementia in 2019 and forecasted prevalence in 2050: an analysis for the Global Burden of Disease Study 2019. Lancet Public Health. 2022;7(2):e105–e125. https://doi.org/10.1016/S2468-2667(21)00249-8 34998485 PMC8810394

[15] Lin SY. “Dementia-friendly communities” and being dementia friendly in healthcare settings. Curr Opin Psychiatry. 2017;30(2):145–150. https://doi.org/10.1097/YCO.0000000000000304 27997454 PMC5287032

[16] Smith K , Gee S, Sharrock T, Croucher M. Developing a dementia-friendly Christchurch: perspectives of people with dementia. Australas J Ageing. 2016;35(3):188–192. https://doi.org/10.1111/ajag.12287 27061350

[17] Lawton MP. Environment and the need satisfaction of the aging. In: Carstensen LL, Edelstein BA, eds. Handbook of Clinical Gerontology. Pergamon Press; 1987:33–40.

[18] Kahana E , Lovegreen L, Kahana B, Kahana M. Person, environment, and person-environment fit as influences on residential satisfaction of elders. Environ Behavior. 2003;35:434–453. https://doi.org/10.1177/0013916503035003007

[19] Auchincloss AH , Mujahid MS, Shen M, Michos ED, Whitt-Glover MC, Diez Roux AV. Neighborhood health-promoting resources and obesity risk (the Multi-Ethnic Study of Atherosclerosis). Obesity (Silver Spring, Md.). 2013;21(3):621–628. https://doi.org/10.1002/oby.20255 23592671 PMC3511654

[20] Choi YJ. Age-friendly features in home and community and the self-reported health and functional limitation of older adults: the role of supportive environments. J Urban Health. 2020;97(4):471–485. https://doi.org/10.1007/s11524-020-00462-6 32601773 PMC7392977

[21] Clarke PJ , Weuve J, Barnes L, Evans DA, Mendes de Leon CF. Cognitive decline and the neighborhood environment. Ann Epidemiol. 2015;25(11):849–854. https://doi.org/10.1016/j.annepidem.2015.07.001 26253697 PMC4609590

[22] Finlay J , Esposito M, Li M, et al Neighborhood active aging infrastructure and cognitive function: a mixed-methods study of older Americans. Prev Med. 2021;150:106669. https://doi.org/10.1016/j.ypmed.2021.106669 34087319 PMC8316307

[23] Choi YJ. Understanding aging in place: home and community features, perceived age-friendliness of community, and intention toward aging in place. Gerontologist. 2022;62(1):46–55. https://doi.org/10.1093/geront/gnab070 34043782 PMC8759482

[24] Finlay JM , McCarron HR, Statz TL, Zmora R. A critical approach to aging in place: a case study comparison of personal and professional perspectives from the Minneapolis Metropolitan Area. J Aging Soc Policy. 2021;33(3):222–246. https://doi.org/10.1080/08959420.2019.1704133 31856684

[25] Flatt JD , Liptak A, Oakley MA, Gogan J, Varner T, Lingler JH. Subjective experiences of an art museum engagement activity for persons with early-stage Alzheimer’s disease and their family caregivers. Am J Alzheimers Dis Other Demen. 2015;30(4):380–389. https://doi.org/10.1177/1533317514549953 25216658 PMC4362745

[26] Phinney A , Kelson E, Baumbusch J, O’Connor D, Purves B. Walking in the neighbourhood: performing social citizenship in dementia. Dementia (London, England). 2016;15(3):381–394. https://doi.org/10.1177/1471301216638180 27170588 PMC5751851

[27] Young R , Camic PM, Tischler V. The impact of community-based arts and health interventions on cognition in people with dementia: a systematic literature review. Aging Mental Health. 2016;20(4):337–351. https://doi.org/10.1080/13607863.2015.1011080 25683767

[28] Eekelaar C , Camic PM, Springham N. Art galleries, episodic memory and verbal fluency in dementia: an exploratory study. Psychol Aesth Creat Arts. 2012;6(3):262–272. https://doi.org/10.1037/a0027499

[29] Papastavrou E , Andreou P, Middleton N, Tsangari H, Papacostas S. Dementia caregiver burden association with community participation aspect of social capital. J Adv Nurs. 2015;71(12):2898–2910. https://doi.org/10.1111/jan.12762 26345604

[30] Thijssen M , Daniels R, Lexis M, et al How do community based dementia friendly initiatives work for people with dementia and their caregivers, and why? A rapid realist review. Int J Geriat Psychiatry. 2022;37(2):gps.5662. https://doi.org/10.1002/gps.5662 PMC9299867 34825742

[31] Gaugler JE , Kane RL, Kane RA, Newcomer R. Early community-based service utilization and its effects on institutionalization in dementia caregiving. Gerontologist. 2005;45(2):177–185. https://doi.org/10.1093/geront/45.2.177 15799982

[32] Joling KJ , Janssen O, Francke AL, et al Time from diagnosis to institutionalization and death in people with dementia. Alzheimer's Dement: J Alzheimer's Assoc. 2020;16(4):662–671. https://doi.org/10.1002/alz.12063 PMC7984226 32072728

[33] Health and Retirement Study. HRS Contextual Data Resource restricted datasets. Published online 2020. Accessed March 10, 2025. https://hrs.isr.umich.edu/news/data-announcements/health-and-retirement-contextual-data-resource

[34] National Neighborhood Data Archive. 2003-2017 Publicly Available Data. Published online 2019. Accessed March 10, 2025. https://nanda.isr.umich.edu/

[35] Crimmins EM , Kim JK, Langa KM, Weir DR. Assessment of cognition using surveys and neuropsychological assessment: the Health and Retirement Study and the Aging, Demographics, and Memory Study. J Gerontol Series B: Psychol Sci Soc Sci. 2011;66B(suppl_1):i162–i171. https://doi.org/10.1093/geronb/gbr048 PMC3165454 21743047

[36] Langa KM , Larson EB, Crimmins EM, et al A comparison of the prevalence of dementia in the United States in 2000 and 2012. JAMA Int Med. 2017;177(1):51–58. https://doi.org/10.1001/jamainternmed.2016.6807 PMC5195883 27893041

[37] Chen Y , Tysinger B, Crimmins E, Zissimopoulos JM. Analysis of dementia in the US population using Medicare claims: insights from linked survey and administrative claims data. Alzheimer's Dement: Transl Res Clin Interven. 2019;5(1):197–207. https://doi.org/10.1016/j.trci.2019.04.003 PMC6556828 31198838

[38] Moeller J. *Standards for Outdoor Recreational Areas*. Published online 1965. Accessed March 16, 2023. https://www.planning.org/pas/reports/report194.htm

[39] Dutko P , Ploeg MV, Farrigan T. Characteristics and Influential Factors of Food Deserts. Department of Agriculture, Economic Research Service; 2012. https://www.ers.usda.gov/webdocs/publications/45014/30940_err140.pdf

[40] Ofstedal MB , Weir DR, Chen KT, Wagner J. Updates to HRS Sample Weights. University of Michigan Survey Research Center; https://hrs.isr.umich.edu/publications/biblio/5917

[41] Lee S , Nishimura R, Burton P, McCammon R. HRS 2016 Sampling Weights. Survey Research Center, Institute for Social Research, University of Michigan; 2021. https://hrs.isr.umich.edu/sites/default/files/biblio/HRS2016SamplingWeights.pdf

[42] Besser L. Outdoor green space exposure and brain health measures related to Alzheimer’s disease: a rapid review. BMJ Open. 2021;11(5):e043456. https://doi.org/10.1136/bmjopen-2020-043456 PMC8098949 33941628

[43] Gong Y , Gallacher J, Palmer S, Fone D. Neighbourhood green space, physical function and participation in physical activities among elderly men: the Caerphilly Prospective Study. Int J Behav Nutritr Phys Activity. 2014;11(1):40. https://doi.org/10.1186/1479-5868-11-40 PMC3994572 24646136

[44] Zhou Y , Yuan Y, Chen Y, Lai S. Association pathways between neighborhood greenspaces and the physical and mental health of older adults—a cross-sectional study in Guangzhou, China. Front Public Health. 2020;8:551453. https://doi.org/10.3389/fpubh.2020.551453 33072696 PMC7536577

[45] Artmann M , Chen X, Ioja C, et al The role of urban green spaces in care facilities for elderly people across European cities. Urban Forest Urban Green. 2017;27:203–213. https://doi.org/10.1016/j.ufug.2017.08.007

[46] Demurtas J , Schoene D, Torbahn G, et al European Society of Geriatric Medicine Special Interest Group in Systematic Reviews and Meta-Analyses, Frailty, Sarcopenia, and Dementia. Physical activity and exercise in mild cognitive impairment and dementia: an umbrella review of intervention and observational studies. J Am Med Dir Assoc. 2020;21(10):1415–1422.e6. https://doi.org/10.1016/j.jamda.2020.08.031 32981668

[47] Jia R , Liang J, Xu Y, Wang Y. Effects of physical activity and exercise on the cognitive function of patients with Alzheimer disease: a meta-analysis. BMC Geriatr. 2019;19(1):1–14. https://doi.org/10.1186/s12877-019-1175-2 31266451 PMC6604129

[48] Bedini LA , Labban JD, Gladwell NJ, Dudley WN. The effects of leisure on stress and health of family caregivers. Int J Stress Manag. 2018;25:43–55. https://doi.org/10.1037/str0000072

[49] Oshio T , Kan M. How do social activities mitigate informal caregivers’ psychological distress? Evidence from a nine-year panel survey in Japan. Health Qual Life Outc. 2016;14(1):117. https://doi.org/10.1186/s12955-016-0521-8 PMC4994414 27549086

[50] Twohig-Bennett C , Jones A. The health benefits of the great outdoors: a systematic review and meta-analysis of greenspace exposure and health outcomes. Environ Res. 2018;166:628–637. https://doi.org/10.1016/j.envres.2018.06.030 29982151 PMC6562165

[51] Choi YJ , Crimmins EM, Ailshire JA. Food insecurity, food environments, and disparities in diet quality and obesity in a nationally representative sample of community-dwelling older Americans. Prevent Med Rep. 2022;29:101912. https://doi.org/10.1016/j.pmedr.2022.101912 PMC9326331 35911578

[52] Roberts RO , Geda YE, Cerhan JR, et al Vegetables, unsaturated fats, moderate alcohol intake, and mild cognitive impairment. Dement Geriatr Cogn Disord. 2010;29(5):413–423. https://doi.org/10.1159/000305099 20502015 PMC2889256

[53] Solfrizzi V , Custodero C, Lozupone M, et al Relationships of dietary patterns, foods, and micro- and macronutrients with Alzheimer’s disease and late-life cognitive disorders: a systematic review. J Alzheimer's Dis. 2017;59(3):815–849. https://doi.org/10.3233/JAD-170248 28697569

[54] Netterville L. *Caregiver Nutrition Education Toolkit*. Published online 2020. Accessed March 10, 2025. https://acl.gov/sites/default/files/nutrition/Caregiver-Nutrition-Education-Toolkit-FInal_508.pdf

[55] Goswami S , Korgaonkar S, Bhattacharya K, Rosenthal M. Food insecurity in a sample of informal caregivers in 4 southern US states. Prev Chronic Dis. 2022;19: 220069. https://doi.org/10.5888/pcd19.220069 PMC9390796 35980833

[56] Prince M , Albanese E, Guerchet M, Prina M. Nutrition and Dementia. Alzheimer’s Disease International; 2014. Accessed March 10, 2025. https://www.alzint.org/resource/nutrition-and-dementia/

[57] Lamar KL , Luke JJ. Impacts of art museum-based dementia programming on participating care partners. J Mus Educ. 2016;41(3):210–219. https://doi.org/10.1080/10598650.2016.1193314

[58] Skufca L. Family Caregiver Retail Preferences and Challenges. AARP Research; 2019. https://doi.org/10.26419/res.00336.001

[59] Oh SS , Cho E, Kang B. Social engagement and cognitive function among middle-aged and older adults: gender-specific findings from the Korean Longitudinal Study of Aging (2008–2018). Sci Rep. 2021;11:15876. https://doi.org/10.1038/s41598-021-95438-0 34354162 PMC8342413

[60] Gaugler JE , Jarrott SE, Zarit SH, Stephens MAP, Townsend A, Greene R. Adult day service use and reductions in caregiving hours: effects on stress and psychological well-being for dementia caregivers. Int J Geriatr Psychiatry. 2003;18(1):55–62. https://doi.org/10.1002/gps.772 12497556

[61] Zarit SH , Kim K, Femia EE, Almeida DM, Klein LC. The effects of adult day services on family caregivers’ daily stress, affect, and health: outcomes from the Daily Stress and Health (DaSH) Study. Gerontologist. 2014;54(4):570–579. https://doi.org/10.1093/geront/gnt045 23690056 PMC4155447

[62] Simning A , Orth J, Wang J, Caprio TV, Li Y, Temkin‐Greener H. Skilled nursing facility patients discharged to home health agency services spend more days at home. J Am Geriatr Soc. 2020;68(7):1573–1578. https://doi.org/10.1111/jgs.16457 32294239 PMC7363542

[63] Simning A , Orth J, Temkin-Greener H, Li Y, Simons KV, Conwell Y. Skilled nursing facility-to-home trajectories for older adults with mental illness or dementia. Am J Geriatr Psychiatry. 2022;30(2):223–234. https://doi.org/10.1016/j.jagp.2021.06.013 34284892 PMC8710182

[64] Bayly M , Morgan D, Chow AF, Kosteniuk J, Elliot V. Dementia-related education and support service availability, accessibility, and use in rural areas: barriers and solutions. Can J Aging. 2020;39:545–585. https://doi.org/10.1017/S0714980819000564 31975685

[65] Vipperman A , Savla J, Roberto KA, Burns D. Barriers to service use among dementia family caregivers in rural Appalachia: implications for reducing caregiver overload. Prev Sci. 2022;24:950–960. https://doi.org/10.1007/s11121-022-01479-w 36543967 PMC9771774

[66] Margot-Cattin I , Ludwig C, Kühne N, et al Visiting out-of-home places when living with dementia: a cross-sectional observational study: Visiter des lieux hors du domicile lorsque l’on vit avec une démence: étude transversale observationnelle. Can J Occup Ther. 2021;88(2):131–141. https://doi.org/10.1177/00084174211000595 33745342 PMC8240000

[67] Ma J , Huang H, Liu D. Influences of spatial accessibility and service capacity on the utilization of elderly-care facilities: a case study of the main urban area of Chongqing. Int J Environ Res Public Health. 2023;20(6):4730. https://doi.org/10.3390/ijerph20064730 36981639 PMC10048546

