# Supplementary material for: Access to Neighborhood Amenities and Services and the Risk of 2-Year Nursing Home Placement Among Persons Living With Dementia
Source: Innov Aging. 2025 Feb 13;9(5):igaf011. doi: 10.1093/geroni/igaf011 (PMC12082093; doi:10.1093/geroni/igaf011)
Supplement: igaf011_suppl_Supplementary_Materials [file igaf011_suppl_supplementary_materials.docx]

**Supplementary Material**

**Table S1.** Multinomial logistic regressions estimating the risk of dying relative to aging in place (N = 3,507)

|  | **Model 1:  Park Area** | | | **Model 2:  Food Access** | | | **Model 3:  Social/Cultural Amenities** | | | **Model 4:  Retail Stores** | | | **Model 5:  Social Services** | | | **Model 6:  Home health** | | |
| --- | --- | --- | --- | --- | --- | --- | --- | --- | --- | --- | --- | --- | --- | --- | --- | --- | --- | --- |
|  | RRR |  | *SE* | RRR |  | *SE* | RRR |  | *SE* | RRR |  | *SE* | RRR |  | *SE* | RRR |  | *SE* |
| Tract-level **Park Area** (ref: no park) |  |  |  |  |  |  |  |  |  |  |  |  |  |  |  |  |  |  |
| Less than 12.5% | 0.90 |  | 0.87 |  |  |  |  |  |  |  |  |  |  |  |  |  |  |  |
| 12.5% or more | 0.66 | * | 0.12 |  |  |  |  |  |  |  |  |  |  |  |  |  |  |  |
| Tract-level **Food Access** (ref: low) |  |  |  |  |  |  |  |  |  |  |  |  |  |  |  |  |  |  |
| High |  |  |  | 1.03 |  | 0.10 |  |  |  |  |  |  |  |  |  |  |  |  |
| **Social/Cultural Amenities** in county ^a, b^ |  |  |  |  |  |  | 1.00 |  | 0.01 |  |  |  |  |  |  |  |  |  |
| **Retail Stores** in tract ^a, b^ |  |  |  |  |  |  |  |  |  | 1.00 |  | 0.00 |  |  |  |  |  |  |
| **Social Services** in county ^a, b^ |  |  |  |  |  |  |  |  |  |  |  |  | 0.88 |  | 0.10 |  |  |  |
| **Home Health Services** in county ^a, b^ |  |  |  |  |  |  |  |  |  |  |  |  |  |  |  | 0.90 |  | 0.06 |
| Age | 1.06 | *** | 0.01 | 1.06 | *** | 0.01 | 1.06 | *** | 0.01 | 1.06 | *** | 0.01 | 1.06 | *** | 0.01 | 1.06 | *** | 0.01 |
| Sex (ref: male) |  |  |  |  |  |  |  |  |  |  |  |  |  |  |  |  |  |  |
| Female | 0.64 | *** | 0.63 | 0.64 | *** | 0.06 | 0.64 | *** | 0.06 | 0.64 | *** | 0.06 | 0.65 | *** | 0.21 | 0.64 | *** | 0.06 |
| Race/ethnicity (ref: non-Hispanic White) |  |  |  |  |  |  |  |  |  |  |  |  |  |  |  |  |  |  |
| Non-Hispanic Black | 0.58 | *** | 0.70 | 0.59 | *** | 0.07 | 0.59 | *** | 0.07 | 0.59 | *** | 0.07 | 0.58 | *** | 0.07 | 0.59 | *** | 0.07 |
| Non-Hispanic other | 0.76 |  | 0.23 | 0.76 |  | 0.23 | 0.77 |  | 0.23 | 0.77 |  | 0.23 | 0.76 |  | 0.26 | 0.77 |  | 0.23 |
| Hispanic | 0.47 | *** | 0.07 | 0.48 | *** | 0.07 | 0.48 | *** | 0.07 | 0.57 | *** | 0.07 | 0.48 | *** | 0.06 | 0.49 | *** | 0.07 |
| Education (ref: less than high school) |  |  |  |  |  |  |  |  |  |  |  |  |  |  |  |  |  |  |
| High school | 1.20 |  | 0.13 | 1.19 |  | 0.13 | 1.18 |  | 0.13 | 1.19 |  | 0.13 | 1.18 |  | 0.20 | 1.19 |  | 0.13 |
| More than high school | 1.26 |  | 0.16 | 1.26 |  | 0.16 | 1.26 |  | 0.16 | 1.25 |  | 0.16 | 1.25 |  | 0.23 | 1.27 |  | 0.17 |
| Household asset (decimal; range: 1-10) | 0.99 |  | 0.17 | 0.99 |  | 0.02 | 0.99 |  | 0.02 | 0.99 |  | 0.02 | 0.99 |  | 0.02 | 0.99 |  | 0.02 |
| Insurance (ref: no insurance) |  |  |  |  |  |  |  |  |  |  |  |  |  |  |  |  |  |  |
| Health insurance | 1.15 |  | 0.33 | 1.15 |  | 0.32 | 1.15 |  | 0.32 | 1.16 |  | 0.32 | 1.14 |  | 1.17 | 1.13 |  | 0.32 |
| Long-term care insurance | 1.23 |  | 0.38 | 1.24 |  | 0.38 | 1.24 |  | 0.38 | 1.25 |  | 0.38 | 1.24 |  | 1.94 | 1.23 |  | 0.37 |
| Living arrangement (ref: living alone) |  |  |  |  |  |  |  |  |  |  |  |  |  |  |  |  |  |  |
| Living with a spouse | 0.71 |  | 0.18 | 0.71 |  | 0.18 | 0.72 |  | 0.18 | 0.72 |  | 0.18 | 0.72 |  | 0.33 | 0.72 |  | 0.18 |
| Living with children | 0.97 |  | 0.11 | 0.97 |  | 0.11 | 0.97 |  | 0.11 | 0.97 |  | 0.11 | 0.97 |  | 0.07 | 0.98 |  | 0.11 |
| Living with relatives | 1.04 |  | 0.17 | 1.04 |  | 0.17 | 1.04 |  | 0.17 | 1.05 |  | 0.17 | 1.06 |  | 0.16 | 1.04 |  | 0.17 |
| Living with unrelated adults | 0.92 |  | 0.20 | 0.92 |  | 0.20 | 0.92 |  | 0.20 | 0.92 |  | 0.20 | 0.93 |  | 0.11 | 0.93 |  | 0.20 |
| Marital status (ref: married/partnered) |  |  |  |  |  |  |  |  |  |  |  |  |  |  |  |  |  |  |
| Separated or divorced | 0.83 |  | 0.21 | 0.83 |  | 0.21 | 0.83 |  | 0.21 | 0.83 |  | 0.21 | 0.84 |  | 0.64 | 0.84 |  | 0.21 |
| Widowed | 0.77 |  | 0.20 | 0.78 |  | 0.20 | 0.78 |  | 0.20 | 0.78 |  | 0.20 | 0.78 |  | 0.68 | 0.78 |  | 0.20 |
| Never married | 0.93 |  | 0.33 | 0.93 |  | 0.34 | 0.94 |  | 0.34 | 0.94 |  | 0.34 | 0.93 |  | 1.49 | 0.94 | * | 0.34 |
| Number of caregivers (range: 0-5) | 1.08 | * | 0.04 | 1.08 | * | 0.37 | 1.08 | * | 0.04 | 1.08 | * | 0.04 | 1.08 | * | 0.06 | 1.08 | *** | 0.04 |
| Self-rated health (range: 1 poor - 5 excellent) | 0.79 | *** | 0.03 | 0.79 | *** | 0.29 | 0.79 | *** | 0.03 | 0.80 | *** | 0.03 | 0.79 | *** | 0.05 | 0.79 | *** | 0.03 |
| Hours of care received per week (ref: <14hours) |  |  |  |  |  |  |  |  |  |  |  |  |  |  |  |  |  |  |
| 14 hours or more | 1.76 | *** | 0.17 | 1.76 | *** | 0.17 | 1.76 | *** | 0.17 | 1.77 | *** | 0.17 | 1.77 | *** | 0.25 | 1.77 | *** | 0.17 |
| Urbanicity (ref: urban) |  |  |  |  |  |  |  |  |  |  |  |  |  |  |  |  |  |  |
| Rural | 0.86 |  | 0.10 | 0.88 |  | 0.10 | 0.89 |  | 0.10 | 0.89 |  | 0.10 | 0.90 |  | 0.18 | 0.89 |  | 0.10 |
| Number of nursing home in county ^a, b^ | 1.01 |  | 0.02 | 1.01 |  | 0.02 | 1.02 |  | 0.04 | 1.01 |  | 0.02 | 1.08 |  | 0.07 | 1.07 | * | 0.04 |
| % population in poverty in tract (range: 0-1) | 0.74 |  | 0.29 | 0.72 |  | 0.29 | 0.72 |  | 0.29 | 0.71 |  | 0.28 | 0.73 |  | 0.08 | 0.73 |  | 0.29 |
| State-level Medicaid HCBS spending ^b^ | 1.00 |  | 0.00 | 1.00 |  | 0.00 | 1.00 |  | 0.00 | 1.00 |  | 0.00 | 1.00 |  | 0.00 | 1.00 |  | 0.00 |
| *Constant* | 0.03 | *** | 0.02 | 0.03 | *** | 0.02 | 0.03 | *** | 0.02 | 0.03 | *** | 0.02 | 0.03 | *** | 0.00 | 0.03 | *** | 0.02 |

*Notes*: HCBS = home and community-based services; ref = reference; RRR = relative risk ratio; *SE* = standard error.

^a^ Number per square mile. Top-coded at 99th percentile.

^b^ Adjusted for the older population (age 65 and over) in the geographic area.

* p<.05; ** p<.01; *** p<.001.

Table S1 presents the models predicting the relative risk of dying. Living in a census tract with 12.5% or more park area significantly reduced the risk of dying by 34% (RRR=0.66, p<.05). Other neighborhood amenities and services were not significantly associated with the risk of dying (food access: RRR=1.03, p=.77; social and cultural amenities: RRR=1.00, p=.91; retail stores: RRR=1.00, p=.24; social services: RRR=0.88, p=.09; home health services: RRR=0.90, p=.09).

**Supplementary Material**

**Table S2.** Multinomial logistic regressions estimating the risk of transition to a nursing home and dying relative to aging in place (N = 3,507)

|  | **Transitioning to**  **a Nursing Home** | | |  | **Dying** | | |
| --- | --- | --- | --- | --- | --- | --- | --- |
|  | RRR |  | *SE* |  | RRR |  | *SE* |
| Tract-level **Park Area** (ref: no park) |  |  |  |  |  |  |  |
| Less than 5.5% | 1.09 |  | 0.15 |  | 0.91 |  | 0.09 |
| 5.5% or more | 0.61 | * | 0.13 |  | 0.74 | * | 0.10 |
| Tract-level **Park Area** (ref: no park) |  |  |  |  |  |  |  |
| Less than 6% | 1.07 |  | 0.15 |  | 0.90 |  | 0.09 |
| 6% or more | 0.64 | * | 0.14 |  | 0.75 | * | 0.10 |
| Tract-level **Park Area** (ref: no park) |  |  |  |  |  |  |  |
| Less than 6.5% | 1.07 |  | 0.15 |  | 0.90 |  | 0.09 |
| 6.5% or more | 0.64 | * | 0.14 |  | 0.74 | * | 0.11 |
| Tract-level **Park Area** (ref: no park) |  |  |  |  |  |  |  |
| Less than 7% | 1.07 |  | 0.15 |  | 0.91 |  | 0.09 |
| 7% or more | 0.62 | * | 0.14 |  | 0.71 | * | 0.11 |
| Tract-level **Park Area** (ref: no park) |  |  |  |  |  |  |  |
| Less than 7.5% | 1.05 |  | 0.17 |  | 0.91 |  | 0.09 |
| 7.5% or more | 0.65 | + | 0.15 |  | 0.69 | * | 0.11 |
| Tract-level **Park Area** (ref: no park) |  |  |  |  |  |  |  |
| Less than 8% | 1.06 |  | 0.15 |  | 0.91 |  | 0.09 |
| 8% or more | 0.59 | * | 0.14 |  | 0.69 | * | 0.11 |
| Tract-level **Park Area** (ref: no park) |  |  |  |  |  |  |  |
| Less than 8.5% | 1.06 |  | 0.15 |  | 0.91 |  | 0.09 |
| 8.5% or more | 0.55 | * | 0.14 |  | 0.67 | * | 0.11 |
| Tract-level **Park Area** (ref: no park) |  |  |  |  |  |  |  |
| Less than 9% | 1.07 |  | 0.15 |  | 0.91 |  | 0.09 |
| 9% or more | 0.48 | ** | 0.13 |  | 0.67 | * | 0.11 |
| Tract-level **Park Area** (ref: no park) |  |  |  |  |  |  |  |
| Less than 9.5% | 1.06 |  | 0.15 |  | 0.90 |  | 0.09 |
| 9.5% or more | 0.49 | ** | 0.13 |  | 0.68 | * | 0.12 |
| Tract-level **Park Area** (ref: no park) |  |  |  |  |  |  |  |
| Less than 10% | 1.04 |  | 0.14 |  | 0.89 |  | 0.09 |
| 10% or more | 0.52 | * | 0.14 |  | 0.71 | + | 0.12 |

Notes: ref = reference; RRR = relative risk ratio; *SE* = standard error.

+p<.10; * p<.05; ** p<.01; *** p<.001.
